# It is More than Thought that Counts: the Role of Readiness for Aggression in the Relationship Between Ostracism and Displaced Aggression

**DOI:** 10.1007/s12144-016-9430-6

**Published:** 2016-04-02

**Authors:** Joanna Rajchert, Karolina Konopka, L. Rowell Huesmann

**Affiliations:** 1 Institute of Applied Psychology, The Maria Grzegorzewska University, ul. Szczesliwicka 40, 02-353 Warsaw, Poland; 2 Institute for Social Research, University of Michigan, Ann Arbor, MI 48104 USA

**Keywords:** Ostracism, Social exclusion, Readiness for aggression, Aggressive behavior

## Abstract

Research has shown that ostracism results in aggressive behavior towards the ostracising other, but also causes displaced aggression—aggression directed towards an innocent person. Our study investigated whether displaced aggressive responses to ostracism were increased by three types of aggression proneness (readiness for aggression) based on different mechanisms: emotional-impulsive, habitual-cognitive or personality-immanent. Participants (*n* = 118) played a Cyberball game in which they were either excluded or included, next prepared a hot sauce sample for another person as an indicator of aggression and completed the Readiness for Interpersonal Aggression Inventory. Results showed that ostracism evoked more aggression in participants with high rather than with low emotional-impulsive readiness for aggression. Only this type of readiness moderated the ostracism-aggression relationship indicating that mostly affective mechanisms induce displaced aggressive responses to exclusion.

## Introduction

Ostracism, being ignored or excluded, and rejection, being rejected after initial or anticipated acceptance have powerful consequences at the neuropsychological, emotional and cognitive levels (Williams 2007a). Research has shown that ostracism thwarts the need to belong, reduces self-esteem, feelings of control and the feeling of having a meaningful existence (Zadro et al. 2004), diminishes prosocial behavior (Coyne et al. 2011a), elicits pain (Eisenberger and Lieberman 2004) and distress (Van Beest and Williams 2006), even when ostracism is only observed on video (Coyne et al. 2011b). Ostracized people are also more susceptible to conformity, compliance and obedience (Riva et al. 2014). Rejection also reduces the global perception of life as meaningful (Stillman et al. 2009), initially causes emotional numbness (DeWall and Baumeister 2006) and even a reduction in intelligent thought (Baumeister et al. 2002). However, it is important to note that ostracism and rejection also have different effects on emotional distress (Bernstein and Claypool 2012a; Bernstein and Claypool 2012b) and preventive versus promotive behavior. As Molden et al. (2009) suggest, the variability in effects arise because ostracism and rejection represent, different to some point, social situations. Ostracism is passive, indirect and implicit, while rejection is active, direct and explicit behavior. Ostracism is a form of exclusion that is often described as a “non-behavior” (Williams 2007b), the most indirect, passive and implicit.

### Exclusion Effects on Aggressive Behavior

Exclusion also affects behavior, provoking aggressive reactions (for review see Baumeister et al. 2007 and Leary et al. 2006). For example, participants who were excluded by individual partners or groups in the laboratory or told that they would end up alone later in life subsequently rated the group members or the partner less favorably than participants, who had not been excluded (Buckley et al. 2004; Leary et al. 1995; Twenge et al. 2001).

Ostracism also caused participants to blast another person with higher levels of aversive noise (DeWall et al. 2010; Twenge et al. 2001) and expose the target to other unpleasant stimuli such as hot sauce or an unappealing snack (Ayduk et al. 2008; Chow et al. 2008; Warburton et al. 2006; Wesselmann et al. 2010).

These experimental results converged with the results of studies exploring the effects of exclusion outside the laboratory. An analysis of school shooting incidents indicated that nearly all the teenage perpetrators had felt rejected by their classmates (Leary et al. 2003).

### Readiness for Aggression Role in Ostracism - Displaced Aggression Relationship

However, as Gerber and Wheeler (2009) show in their meta-analysis of experimental studies on exclusion, ostracized participants differ in their aggressive behavior. Studies reviewed by these authors suggest that prosocial acts that foster belonging could also be the reaction to exclusion, especially in situations when restoring control through aggressive actions is impossible. Research suggests, that there are also some individual differences that magnify or constrain the aggressive reaction to exclusion. Earlier studies looked at the influence of narcissism (Bushman and Baumeister 1998; Twenge and Campbell 2003), perceived self-superiority and positive self-appraisals (Kirkpatrick et al. 2002), rejection sensitivity (Ayduk et al. 2008; Buckley et al. 2004) and implicit theories of relationship (Chen et al. 2012). Individuals, who scored highly on those characteristics behaved more aggressively toward someone who excluded them, than individuals with lower narcissism, less self-superiority or less rejection sensitivity. However, in the context of acceptance, these personality characteristics did not affect the intensity of aggressive behavior. The aim of the current study was to investigate further why ostracism sometimes leads to more and sometimes to less aggressive behavior.

We propose that individual differences in aggressive reactions to ostracism are based either on emotional, cognitive or motivational mechanisms that can regulate aggressive behavior. Most contemporary aggression theories define a set of underlying mechanisms through which personality variables influence aggressive behavior (Anderson and Bushman 2002; Berkowitz 1984; Crick and Dodge 1994; Huesmann 1988; Slotter and Finkel 2011). These underlying mechanisms encompass cognitive processing, negative effect, self-regulation and social-information processing. Berkovitz (e.g., 1993) proposed that frustration or other aversive events produce negative affect, which stimulates thoughts, memories, physiological and motor reactions that might be associated with anger leading to aggressive behavior. According to Cognitive Neoassociation Theory aggressive thoughts, emotions and behavior are linked together in memory and activation of one aggressive concept is spread to other associated concepts. Readily accessible aggression-related cognitions play a particularly salient role in Huesmann’s script theory (1998), which emphasizes the reciprocal connection between cognitive scripts and negative affect or anger. Social interaction theory (Tedeschi and Felson 1994) on the other hand, emphasizes the role of motivation in aggressive behavior. Aggression is perceived in this theory as social influence behavior that allows for obtaining one’s goals (e.g., boosting self-esteem). The social-information processing theory (Crick and Dodge 1994) also emphasizes the cognitive mechanism lying at the base of aggressive behavior, namely the hostile attributions bias. The General Aggression Model (Anderson and Bushman 2002) built on the above mentioned domain specific theories of aggression also underline the role of cognitive structures (schemata and scripts), which are linked to or contain affective states and together influence perception, interpretation, decision making and action. Clearly then, aggression theories have implicated at least two mechanisms in the production of aggressive reactions.

In a deeper exploration of processes that lead to aggressive behavior, Frączek (2002) formulated the idea of “readiness for aggression” (RA). Readiness for aggression is broadly defined as a constellation of psychic processes and structures that underlie and regulate aggressive behavior. Three patterns of RA—Emotional-Impulsive Readiness (E-IR), Habitual-Cognitive Readiness (H-CR) and Personality-Immanent Readiness (P-IR) were distinguished theoretically (Frączek 2002; Frączek et al. 2016) and the three-factor structure of the RA concept was verified empirically (Frączek et al. 2009).

Emotional-Impulsive Readiness refers to an individual’s propensity to react with anger to provocation or frustration and is associated with low emotional control. Defined in this way E-IR is closely related to the constructs of trait anger, emotional reactivity and impulsivity, but also to trait aggressiveness (Konopka et al. 2009; Smulczyk 2008; Smulczyk et al. 2009). In this case the aggressive response is a manifestation of a negative emotional state; it is impetuous and limited in time.

The second pattern of RA, H-CR describes a pattern of habits, scripts and beliefs related to aggressive behavior. This class of RA is related to instrumental aggression; cognitive schemata for aggression may be activated by provocation, the aggressive behavior is habitual and the individual typically approves of aggression, believing that it is desirable to behave aggressively in certain situations (Frączek 2002; Frączek et al. 2016). The studies cited above showed that the H-RA construct was not related to temperamental emotional reactivity or emotionality, and the correlation with impulsivity was weak. There was no significant correlation with neuroticism, although the association between H-CR and psychoticism was significant. Habitual-cognitive readiness was also correlated with physical aggression, verbal aggression and hostility, but not with anger.

A third type of RA, P-IR captures motivational factors related to aggression. It was defined as a stable desire to hurt others in order to obtain pleasure and satisfaction. In theoretical terms P-IR is associated with proactive, need-oriented and spontaneous forms of aggression, which may occur with or without provocation. This type of RA was strongly associated with Eysenck’s psychoticism factor, but also with trait aggressiveness. There was also a moderate association between P-IR and physical and verbal aggression, and weaker associations with anger and hostility.

According to the assumption set out in the General Aggression Model (Anderson and Bushman 2002) that aggressive behavior is regulated by multiple motives, it was confirmed that the different RA types are correlated with each other (Frączek et al. 2009; Frączek et al. 2016; Frączek et al. 2013). The strongest relationship was found between H-CR and P-IR, and the weakest correlation was between E-IR and H-CR.

### Current Study

Displaced aggression occurs when a provoked person is prevented from retaliatory action against the provocateur and subsequently behaves aggressively against an innocent target (Dollard et al. 1939). Existing results show that ostracism as well as rejection lead to more displaced aggression (DeWall et al. 2010; Twenge et al. 2001; Warburton et al. 2006) and that this relationship is stronger for narcissists (Twenge and Campbell 2003). DeWall et al. (2009) also confirmed that the hostile cognitive bias among excluded participants was related to their aggressive treatment of others, who were not involved in the exclusion experience and others with whom participants had no previous contact.

We were interested in displaced aggression and not direct aggression, partly because displaced aggression has not yet been fully explored, especially in the context of ostracism, but mostly because it was an effective way of investigating the role of different RA patterns in the relationship between ostracism and aggression. In a theoretical model of triggered displaced aggression, Miller et al. (2003) assumed that the processes underlying displaced aggression were based on affective arousal elicited by the provocation (in our study the impelling factor was the ostracism).

If affective arousal would be the most prominent factor leading to displaced aggression after ostracism, than we could predict that a higher propensity for emotional-impulsive aggression would boost the displaced aggression among ostracized participants. Drawing on previous studies, Leary et al. (2006) proposed a number of explanations for the association between rejection and aggression. Some of those explanations related mainly to affective and self-control processes, for example rejection as a source of pain or frustration, and a cause of ego-depletion increases negative affect and at the same time diminishes the ability for impulse control leading to outbursts of aggression directed at anybody, even those not involved in the ostracism situation. These explanations are in line with our prediction that high E-IR is a moderator of ostracism - displaced aggression link.

However, in another of Leary et al.’s (2006) explanations, aggression was the outcome of intentions to regain control or social influence, or teach others a lesson. The important role of need for control over a situation was also stressed in Gerber and Wheeler’s (2009) meta-analysis of rejection effect on aggression. Yet another explanation framed the pleasure the individual may take in aggressive acts in terms of relief from anger.

The above explanations also suggest that cognitive and motivational mechanisms may play a role in the ostracism - aggression relationship. In that case, high H-CR people, with more developed aggressive scripts, habitually aggressive and approving of aggression, should behave aggressively in many social situations. But the question is whether H-CR would moderate aggressive behavior after ostracism directed toward an innocent other—displaced aggression. If the aggressive behavior is directed with the aim of regaining the sense of control over the situation or social influence, would the behavior still be effective in satisfying these needs when the ostracizing target is not present?

We can assume that experiencing ostracism leads to the activation of a pre-existing hostile cognitive bias. DeWall et al. (2009) argued that this cognitive bias is the main mechanism underlying the association between rejection, and direct as well as indirect aggression. People with high H-CR, having more developed aggressive scripts, should react more aggressively toward innocent others because ostracism activates hostile cognitions that influence perception and decision making. However, it remains questionable whether the displaced aggression after ostracism would be affected not only by E-IR but also by H-CR. With reference to P-IR, one might hypothesize that it could be associated with more aggressive behavior toward an innocent other regardless of the ostracism context, because aggression in high P-IR people is a source of satisfaction and pleasure and as such can also be generated without any provocation.

We do not wish to imply that affective, cognitive or motivational processes are mutually exclusive or independent mechanisms for producing aggressive behavior. Rather we suggest that each person can be characterized by three patterns of RA which altogether define that person’s aggressive proclivity, however a specific factor may be more or less dominant. Focusing on the relation between ostracism and displaced aggression, rather than direct aggression might allow for showing differences between the three types of readiness for aggression.

#### Hypothesis

We predicted, that while (1) E-IR should moderate the ostracism - displaced aggression link intensifying aggression, and (2) P-IR should inflate aggression scores independently of experimental conditions, (3) the role of H-CR is more unclear and having the target innocent might limit the moderating effect of this RA type (although there are reasons to predict the moderation).

## Method

### Participants and Design

One hundred and twenty-three undergraduates were enrolled in the study in exchange for course credits. The final number of participants was smaller: one participant refused to take part in the hot sauce allocation procedure—the index of aggressive behavior—and the hot sauce allocations of four participants were dropped from the experiment sample because they were considered outliers (at least 2 *SD* from the mean in each condition). Ultimately the sample consisted of 118 students aged between 18 and 22 years old (mean age 18.91, *SD* = .86). There were 45 men (38 %) and 73 women (62 %) in the experiment. Participants were randomly assigned to the exclusion (*n* = 64; 26 men and 38 women) or the inclusion condition (*n* = 54; 19 men and 35 women).

### Procedure and Measures

Participants first gave details about their sex, age and rated their mood on nine-point scale by answering “how they were feeling in this moment” in terms of feeling bad - good, sad - happy, tensed - relaxed, not at all excited - excited, not at all hurt - hurt, not all angry - angry. A similar method of mood measurement was used in other studies on ostracism (e.g., Buckley et al. 2004; Warburton et al. 2006).

Next, participants played the Cyberball game (Williams and Jarvis 2006) which was presented by the experimenter as a form of mental visualization training. In fact the game was intended to induce a feeling of exclusion or inclusion in participants. Participants were told that they would be playing an online game of ball toss with two other participants who were somewhere else, and who they would not meet in person. In the inclusion condition the participant acquired 33 % of all tosses and in the ostracism condition only the first two tosses out of 30 were directed to the participant.

After the Cyberball game, participants completed the manipulation check items, again completed the mood scales and were told that the study had finished. Next they were asked whether they would help with another study entitled “taste sensitivity”. This was used to obtain a measurement of aggression using the hot sauce procedure developed by Lieberman et al. (1999) and adapted by Warburton et al. (2006). Participants were asked to prepare a hot sauce taste sample for a tester who was not involved in the Cyberball game but would be taking part in the taste sensitivity study. The participants learned from the written instruction that they could allocate as much or as little of the sauce as he or she chose, but that the tester would always have to eat the whole sample and that the taster did not like the hot-spicy food (in the taste preferences questionnaire the hot-spicy taste was rated “3” on a 21-point scale).

When the sample had been prepared the experimenter presented participants with the RA inventory.^1^ After completion of the RA inventory, participants were asked to specify on a 21-point scale “How did the tester like hot-spicy food?” With the completion of that item, the study ended and the participants were thanked and debriefed.

#### The RA Measurement

*The Readiness for Interpersonal Aggression Inventory* (RIAI; Frączek et al. 2009; Frączek et al. 2013) consists of three subscales each measuring one type of RA: E-IR (sample item “I have sudden angry outbursts”), H-CR (sample item “I think that some people don’t deserve to be treated very nicely”), and P-IR (sample item “I sometimes feel like hurting someone without any obvious reason”). Each subscale consists of 10 statements with “yes” and “no” response options. The RIAI was developed using exploratory factor analysis (EFA) on a sample of 1233 Polish participants. Temporal and internal stability was good; in a series of measurements with a six-month interval r fell between .55 and .76 and α between .67 and .77. In the current sample an English version of the RIAI was used. The RIAI was translated from Polish to English by a native English speaker. Following this a back-translation was made. As a final step the English version was proofread by a native American English speaker. Cronbach’s alpha indicated that subscale reliability was acceptable for E-IR, α = .65; good for H-CR, α = .76 and poor, although still acceptable for P-IR, α = .52 (Kline 2000). Item elimination did not improve the reliability.

## Results

### Manipulation Check

To demonstrate that the ostracism manipulation was successful we compared self-report data on feeling excluded and percentage of ball tosses directed to participants for the two experimental conditions. On a seven-point scale participants in the ostracism condition reported feeling less included (*t*(117) = −17,39, *p* < .001, *M* = 2.07, *SD* = 1.30) than included participants (*M* = 6.77, *SD* = 1.58, *d* = −3.26), and reported that they had received a smaller percentage of ball tosses from their game partners (*t*(117) = −14.32, *p* < .001, *M* = 8.38, *SD* = 10.31) than included participants (*M* = 39.87, *SD* = 13.10, *d* = −2.69). Results showed that participants had noticed that the taster did not like hot and spicy food; the average of their rating for the taster’s preference for hot-spicy tastes was 4.01 (*SD* = 2.74), and there was no group difference (*t* < 1).

### The Effect of Ostracism on Mood

We also tested the differences in mood and emotions due to ostracism manipulation using repeated measures ANOVA. The results are presented in Table 1.

**Table 1:** Differences in means (M) and standard deviations (SD) of mood indices across conditions and time of measurement

	Time 1 M/SD		Time 2 M/SD		*F*	*p*	*η* ^*2*^ *p*
	Included	Ostracized	Included	Ostracized			
Bad - good	6.50/1.37	6.49/1.25	6.64/1.19	4.49/1.72	30.86	< .001	.21
Sad - happy	6.51/1.45	6.39/1.28	6.51/1.25	5.55/1.66	12.69	.001	.10
Tensed - relaxed	6.22/1.58	5.82/1.66	6.51/1.74	5.38/1.85	8.38	.005	.07
Excited	4.66/1.45	4.84/1.47	4.61/1.44	3.76/1.70	18.50	<.001	.14
Angry	1.68/1.15	2.11/1.20	1.72/1.12	3.23/1.98	24.60	<.001	.18
Hurt	2.03/1.62	2.20/1.39	1.83/1.24	3.19/1.86	22.50	<.001	.16

Results show that participants differed in all measured mood items between conditions after manipulation (all post-hoc tests were significant, *t* > 3.48 and *p* ≤ .001) but not before manipulation (*t* < 1.35 and *p* > .18). What is more, ostracized participants declared more negative and less positive feelings (felt worse, less happy and more sad, less excited, more angry and hurt) than included participants.

We also tested whether time 2 anger or other mood indices and also mood change, operationalized as the difference between time 1 and time 2 mood ratings, mediated the effect of ostracism on aggression. The results of Sobel’s tests showed that none of the 12 mediation effects was significant (for example the indirect effect for time 2 anger was *b* = − 0.20; *SE* = 0.13; *z* = −1.76; *p* > .05).

### The Moderating Role of Readiness for Aggression

The aggression scores were skewed (Kolmogorov-Smirnov test was significant *p* < .001), so we conducted the square root transformation of the data, which normalized the distribution. All presented further analysis were conducted with transformed aggression scores. Before testing the main moderation hypothesis we conducted some preliminary analyses. First we performed the correlation analysis between RA, aggression and all mood indices before and after manipulation. In Table 2 we showed correlations between aggression, RA and anger (before Cyberball - anger 1 and after Cyberball - anger 2) as it was the most closely related to other study variables.

**Table 2:** Means, SD and zero-order correlations between studied continuous variables

	M/SD	Anger 1	Anger 2	E-IR	H-CR	P-IR	DA
Anger 1	1.89/1.20	-	.72***	.20*	.17	.12	.17
Anger 2	2.55/1.80		-	.19*	.08	.10	.32***
E-IR	13.78/2.14			-	.23*	.17	.26*
H-CR	12.38/2.28				-	.42***	.26*
P-IR	11.21/1.32					-	.22*
DA	3.17/1.38						-

E-IR - emotional-impulsive readiness for aggression; H-CR - habitual-cognitive readiness for aggression; P-IR - personality-immanent readiness for aggression; DA - displaced aggression * *p* < .05, ** *p* < .01, *** *p* < .001

Next we tested the differences in displaced aggression due to ostracism versus inclusion and sex differences in RA patterns and displaced aggression (means and standard deviations are included in Table 3).

**Table 3:** Sex differences in readiness for aggression and displaced aggression

	Males	Females			
	*M*/SD	*M*/SD	t-test (117)	*p*	Cohen’s *d*
E-IR	13.77/2.15	13.78/2.16	−.00	.994	.00
H-CR	13.48/2.71	11.71/1.66	3.95	.000	.84
P-IR	11.93/1.49	10.78/.98	5.05	.000	.97
DA	3.43/1.50	3.01/1.29	1.62	.106	.30

E-IR - emotional-impulsive readiness for aggression; H-CR - habitual-cognitive readiness for aggression; P-IR - personality-immanent readiness for aggression; DA - displaced aggression

Analysis showed that all RA patterns were correlated except for E-IR and P-IR. The most closely related were H-CR and P-IR, but there were weaker associations between E-IR and H-CR. Baseline (time 1) and time 2 anger were positively and significantly correlated with E-IR, but only time 2 anger was significantly related to aggression. We also did correlation analysis for other mood indices, which showed that baseline tensed - relaxed mood was negatively related to aggression (*r* = −.216, *p* = .019) and all mood indices measured after manipulation were correlated with aggression in the predicted direction, which was not surprising considering the above reported repeated measures ANOVA results. All three patterns of RA were also positively related to displaced aggression. As predicted, aggressive behavior was dependent on experimental condition (*t*(117) = 5.42, *p* < .001; *d* = .98). Excluded participants were more likely to aggress against innocent others (*M* = 3.72, *SD* = 1.52) than included (*M* = 2.52, *SD* = 0.82).

Similar to previous studies, women were lower in H-CR and P-IR than men (Frączek et al. 2013; Frączek et al. 2016), but we did not observe significant sex differences in E-IR or aggression.

Next we tested the critical moderation hypothesis in a multiple regression by including in one model the three standardized (zero-centered) indices of RA, time 2 anger, experimental conditions, sex and interactions between each RA pattern and ostracism versus inclusion as predictors of the amount of hot sauce allocation to the innocent other (Table 4).

**Table 4:** Regression coefficients for model predicting amount of hot sauce allocation for the third party out of readiness for aggression and experimental conditions controlling for sex and time 2 anger

Predictors				
	*B*	*SE*	β	*p*
Constant	3.82	0.41	-	.000
Anger time 2	0.05	0.12	0.04	.671
Sex (ref. female)	−0.04	0.24	−0.01	.881
Inclusion/ostracism (ref. ostracism)	−1.23	0.24	−0.45	.000
E-IR	0.63	0.16	0.46	.000
H-CR	0.19	0.15	0.14	.206
P-IR	0.27	0.18	0.20	.147
E-IR x Inclusion/ostracism	−0.51	0.22	−0.28	.022
H-CR x Inclusion/ostracism	−0.30	0.26	−0.12	.240
P-IR x Inclusion/ostracism	−0.11	0.23	−0.06	.638

E-IR - emotional-impulsive readiness for aggression; H-CR - habitual-cognitive readiness for aggression; P-IR - personality-immanent readiness for aggression

Results showed that sex and anger were not significant predictors of aggression. Further, we observed that more hot sauce was allocated to innocent third parties by participants with higher E-IR irrespective of the experimental conditions. Consistent with our hypothesis, the interaction term was significant for E-IR even when we controlled for H-CR, P-IR and their interactions with ostracism.

**Fig. 1:**
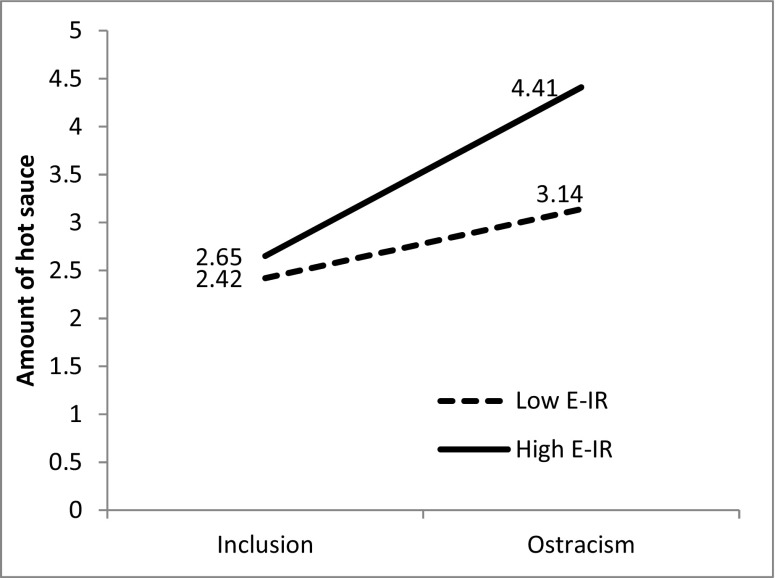
Amount of hot sauce (root squared transformed) allocated as a function of experimental condition and E-IR plotted at −1 SD (low E-IR) and +1 SD (high E-IR) controlling for other variables in the model

We also tested the model excluding insignificant sex and anger predictors. The interaction between experimental conditions and E-IR was then even more significant (*p* = .014), but we decided to present the full model as readers could be interested in the detailed effects of variables (sex, anger) which are often explored in aggression and ostracism studies.^2^ Simple slope analysis (Aiken and West 1991) showed that the effect of exclusion on aggression was strong and positive for high E-IR participants (*b* = −1.75, *SE* = 0.34, *p* < .001), but not that strong, although still significant for low E-IR participants (*b* = −0.71, *SE* = 0.34 *p* = .022), see Fig. 1.

Additionally, there was a positive association between E-IR and sauce allocation in the exclusion condition (*r*(64) = .44, *p* < .001), but the relation was not significant in the inclusion condition (*r*(54) = .16, *p* = .227).

Two other interaction terms turned out to be not significant (*p* > .240).

Anger was not a significant mediator in the ostracism - aggression relationship and was responsible only for a small part of the interactive E-IR and ostracism effect on aggression. However our study allowed us to test the theoretical assumption that E-IR intensifies anger after ostracism. To verify our prediction we used multiple regression analysis in which we included sex, all RA patterns and their interactions with ostracism manipulation as predictors of time 2 anger. The model was presented in Table 5.

**Table 5:** Regression coefficients for model predicting time 2 anger out of readiness for aggression and experimental conditions controlling for sex

Predictors				
	*B*	*SE*	β	*p*
Constant	2.90	.58	-	.000
Sex (ref. female)	.29	.34	.08	.397
Inclusion/ostracism (ref: ostracism)	−1.64	.29	−.45	.000
E-IR	.73	.22	.40	.001
H-CR	−.30	.21	−.16	.164
P-IR	.42	.26	.23	.104
E-IR x Inclusion/ostracism	−.71	.30	−.30	.021
H-CR x Inclusion/ostracism	.67	.36	.20	.063
P-IR x Inclusion/ostracism	−.27	.33	−.10	.412

E-IR - emotional-impulsive readiness for aggression; H-CR - habitual-cognitive readiness for aggression; P-IR - personality-immanent readiness for aggression

**Fig. 2:**
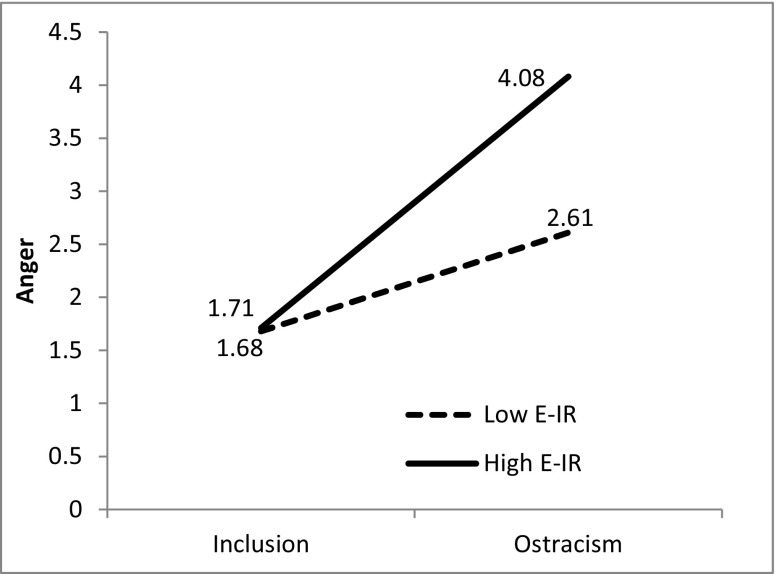
Anger as a function of experimental condition and E-IR plotted at −1 SD (low E-IR) and +1 SD (high E-IR) controlling for other variables in the model

Results showed that the main effect of ostracism manipulation and E-IR as well as the effect of E-IR and ostracism interaction were significant predictors of time 2 anger.^3^ Simple slopes analysis revealed that anger scores differed more between conditions for high E-IR (*b* = −2.36; *SE* = 0.42; *p* < .001) than for low E-IR participants (*b* = −0.92; *SE* = 0.42; *p* = .031). Ostracized participants with high E-IR declared the most intensive angry feelings (see Fig. 2).

## Discussion

According to the General Aggression Model (Bushman and Anderson 2001), the outcome elicited by situational variables related to aggression should vary with dispositional variables. Our study showed that the effect of exclusion on displaced aggression was moderated by only one internal type of RA. We predicted that ostracism would increase aggressive behavior more among individuals high on E-IR—who have a stronger tendency to experience anger and lower emotional control than low E-IR individuals. This prediction was confirmed. The results of this study were consistent with reports relating aggression to emotional susceptibility, irritability, trait anger and impulsiveness (Caprara et al. 1986; Godlaski and Giancola 2009).

Our study showed that ostracized participants experienced more negative affect. Previous studies (e.g., Baumeister et al. 2002; Buckley et al. 2004; Twenge et al. 2001) which explored the role of negative emotions in the relationship between exclusion or rejection and aggression indicated that negative affect, although influenced by exclusion, did not mediate the effect of exclusion on aggression. However, Chow et al. (2008) suggested that the focus should be on anger not general negative affect and showed that anger but not sadness mediated the effect of ostracism on aggression. In our study, ostracized participants also felt angrier than included individuals, and anger was positively related to aggressive behavior. Nevertheless, the mediation effect was not significant, pointing to other processes that take place in the relationship between ostracism and aggression.

Results showed that anger is more readily elicited by ostracism in high E-IR individuals, and it seems that in combination with the low self-control characteristic of this RA pattern, ostracism produced more aggressive behavior in high E-IR participants. However, although E-IR was related to more anger before and after ostracism, controlling for time 2 anger did not change the interaction effect substantially, showing that E-IR impact on aggression is only in some part affected by anger.

One consequence of ostracism may be particularly important for understanding the role of E-IR in the relationship between exclusion and aggression. A high E-IR means not only high proneness to anger but also low self-control. In other words, the self-control strength of high E-IR individuals is weak on an everyday basis. The strength model of self-control posits that situations, such as exclusion, impair or deplete self-control (Muraven and Baumeister 2000) which is a limited resource. Previous attempts to self-control negative internal states evoked by exclusion lead to failure in consecutive self-control attempts. As high E-IR individuals exhibit low self-control strength even in neutral situations, an ostracism incident deprived them of control more easily than others, leading to un-controlled aggressive behavior. It is also possible, based on Gerber and Wheeler’s (2009) observations that the need for control among high E-IR individuals is more affected by ostracism compared to others and thus they strive more for its restoration by being more aggressive.

We hypothesized that H-CR participants would also show higher displaced aggression, as ostracism activates the hostile cognitive bias, which would influence the perception and decision making of the individual leading to higher aggression even toward an innocent other. Berkowitz (2008) emphasized that frequent activation of an aggression script (corresponding to high H-CR) results in aggressive behavior, which occurs immediately after the triggering event. Previous studies have shown that when a hostile cognitive bias is activated, individuals have a tendency to perceive aggression as quite typical of social interactions and to perceive neutral information and ambiguous actions by another person as hostile (Bushman and Anderson 2001; DeWall et al. 2009). So it should be possible that ostracism causes a hostile cognitive bias, along with angry thoughts (Berkowitz 2012) thus facilitating more aggressive behavior among high H-CR individuals who have many well developed aggressive scripts.

However, we also postulated that aggressive behavior in high H-CR participants is based on norms and beliefs that approve aggression in certain situations in which it can be instrumental. In other words, high H-CR participants should be more aggressive when such behavior allows for gaining one’s goals, such as regaining control or teaching somebody a lesson. From this point of view aggression toward someone, who was not involved in ostracism of the target would not be effective and would not meet the standard for approved aggressive behavior.

Results showed that the behavior of individuals with high H-CR was consistent with a norm permitting aggression only when zero-order correlations were taken into account and other variables were not controlled. When we controlled for other variables, H-CR did not influence aggressive behavior after ostracism.

Although it is risky to discuss the lack of a relationship, as the reasons behind it could be multiple and diverse (statistical, e.g., not enough statistical power to detect differences, when the effect is small; methodological, e.g., measurement flaws or theoretical), we propose some theoretical explanations.

Berkowitz (1987, 1990) assumed that the frustrating or provoking episode elicits negative affect first, which in turn activates hostile cognitions, consequently leading to overt aggression. However, this automatic reaction might be suppressed. According to Berkowitz (1990), an aggressive reaction depends on cognitions about the cause of negative feelings. The perception of responsibility for the aversive event is a crucial determinant of external manifestations of anger (Berkowitz and Harmon-Jones 2004). While exclusion might foster negative affect along with hostile cognitions, aggressive behavior is an effect of further cognitive processing of the situation.

Also, Robinson et al. (2010) proposed that cognitive control plays an important role in behavior regulation and referred to studies showing that activation of hostile thoughts do not always lead to aggression. The context of the situation is important and people recruit cognitive control wisely, in particular, for situations in which not doing so would be problematic. Bartholow et al. (2005) also showed the important role of hostile cognition and context in aggression, although from a different angle. In this study, weapons priming effect on aggression depended on the content of the aggressive script; hunters related weapons with hunting, not with aggression, thus priming was not effective with hunters.

High H-CR individuals posses well developed cognitive scripts for aggressive behavior. However, it seems that this cognitive type of readiness for aggression does not refer to the general approval of aggression in all possible situations, but rather operates in specific circumstances, which are included in particular aggressive script (specific situations, e.g., “I think it is necessary to be violent in some situations,” and toward people who are responsible for the wrong doing, e.g., “You have to teach a ruthless lesson to those who deserve it”). In other words, aggressive behavior of high H-CR individuals may be performed automatically, but only when the triggering cue is available and was previously incorporated into the aggressive schema. Violence toward the innocent other is generally regarded as non-normative and does not fulfill any criteria that allows one to behave aggressively, thus does not constitute the content of an aggressive script even among high H-CR individuals.

Finally, P-IR reflecting a stable desire to hurt others independently of social context, was expected to be related to increased aggression in both experimental conditions. Correlation results were encouraging as P-IR was positively related to aggressive behavior when no other RA was controlled. However, in the tested regression model, the motivational component of RA failed to predict hot sauce allocations. A probable explanation is that the internal reliability of P-IR subscale in the US sample was not satisfactory. Maybe the three-factor structure of the Polish version of the RIAI cannot be replicated in a US sample because of cultural differences; therefore a factor analysis including more participants is necessary. Another possible explanation, related to the problem of the factor structure of the RIAI was that P-IR in our study shared a lot of variance with H-CR, which did not predict aggression either.

Nevertheless, further research is needed to improve the P-IR scale’s reliability and establish the factor structure of the RIAI in the US. The cultural background of Poland and the USA is different with Poland being less individualistic (Hofstede 1980; Oyserman et al. 2002), and research shows that there are cultural differences in the readiness for aggression measured by the RIAI (Frączek et al. 2013, 2016). Also, further studies would be useful in which the conditions for H-CR influence could be tested by the manipulation of the aggression target participation in the ostracism-inclusion incident.

## Conclusions

There are many studies on moderators of the relationship between ostracism and aggression. Researchers have mostly examined individual differences such as narcissism or trait self-esteem in this respect. However, there are few reports dealing with the individual differences in the propensity to act aggressively based on different mechanisms. In our study, we explored a new concept of readiness for aggression and showed that mainly emotional-impulsive readiness resulted in displaced aggression. On the other hand, the influence of habitual-cognitive readiness on the ostracism-aggression link probably requires more specific situational conditions in which an aggressive response is allowed or even demanded, such as aggression target participation in the ostracism incident. By illuminating the role of emotional-impulsive tendencies to aggress as a moderator of ostracism effect on displaced aggressive behavior, our study indicated that in order to effectively prevent the antisocial results of ostracism, we would need to pay more attention to self-control and affect regulation processes.

## Footnotes

1 The RA inventory includes items related to aggressive behavior that could serve as signals of aggressive behavior (Berkowitz 1989) and influence the measurement of aggression that followed Cyberball in all or only in some participants. To avoid priming aggression with the RA questionnaire, the measure was administrated after the hot sauce procedure. For this reason it was necessary to demonstrate that RA was not influenced by exclusion vs. inclusion. Analysis showed that there were no differences between the excluded and included group in terms of RA (*p* between 0.413 for H-CR and 0.614 for E-IR); nor were the RA means different from RA means obtained in a correlation study conducted on a very similar group of participants derived from the same participant pool (*p* between 0.150 for E-IR and 0.599 for H-CR); we could then assume that Cyberball did not affect RA indices.

2 We also tested the model controlling for the change in anger (difference between time 1 and time 2 anger); the results were similar (e.g., E-IR x Condition interaction term *β* = −.29; *p* = .012).

3 We also conducted analysis controlling for time 1 anger; as time 1 anger is correlated with E-IR, the main effect of E-IR and the E-IR x Condition effect were no longer significant; the main effects of variables less related to baseline angry feelings were demonstrated, namely H-CR (*β* = −.19; *p* = .025) and P-IR (*β* = .20; *p* = .050) and the effect of condition was still present (*B* = −.32; *p* < .001); almost identical results were demonstrated when we predicted a change in anger; H-CR predicted a reduction in anger and PIR predicted an increase in anger independently of experimental manipulation; there was no significant interaction effect observed.
